# Reactive oxygen species mediate anlotinib-induced apoptosis via activation of endoplasmic reticulum stress in pancreatic cancer

**DOI:** 10.1038/s41419-020-02938-4

**Published:** 2020-09-17

**Authors:** Liguo Yang, Xiaoshu Zhou, Jinrui Sun, Qianghui Lei, Qi Wang, Di Pan, Mingxing Ding, Yi Ding

**Affiliations:** 1 College of Veterinary Medicine, Huazhong Agricultural University, Wuhan, China; 2 Cancer Center, Union Hospital, Tongji Medical College, Huazhong University of Science and Technology, Wuhan, China

**Keywords:** Targeted therapies, Drug development

## Abstract

Anlotinib (AL3818), a novel multi-targeted receptor tyrosine kinase inhibitor, has recently been proven to be an antitumour drug. This study aimed to explore the antitumour effect of anlotinib and its underlying molecular mechanisms in human pancreatic cancer (PC) cells. The anti-proliferative effect of anlotinib for three PC cell lines was validated using CCK-8, colony formation and EdU detection assays. Cell cycle, cell apoptosis, and reactive oxygen species (ROS) detection assays, a PC xenograft model and immunohistochemistry were performed to elucidate the mechanisms by which anlotinib induced tumour lethality in vitro and in vivo. These results demonstrated that anlotinib inhibited proliferation, induced G2/M phase arrest and triggered apoptosis in PC cell lines. Anlotinib induced PC’s apoptosis through the accumulation of ROS which activated the endoplasmic reticulum (ER) stress via PERK/p-eIF2α/ATF4 pathway. Furthermore, we demonstrated that the expression level of Nrf2, an antioxidant protein, increased with anlotinib treatment. Nrf2 knockdown enhanced the pro-apoptotic effect of anlotinib and the expression of the PERK/p-eIF2α/ATF4 pathway. The in vivo results suggested that suppressing Nrf2 improved the antitumour effect of anlotinib on PC cells. These data indicated that the apoptotic effect of anlotinib on PC cells was induced by ER stress via the accumulation of ROS. In the future, anlotinib combined with an Nrf2 inhibitor may provide a new therapeutic strategy for the treatment of human PC.

## Introduction

Pancreatic ductal adenocarcinoma (PDAC) results in more than 90% of all types of pancreatic cancers (PC) and is predicted to be the third leading cause of cancer death in the next decade^1,2^. Despite progress in treatments, patients with PDAC respond poorly to chemotherapy, mainly due to drug resistance. First-line chemotherapy with gemcitabine^3^ or the FORFIRINOX regime (5-fluorouracil, leucovorin, irinotecan, and oxaliplatin)^4^ has only a 5.4% partial response rate in patients with PDAC. Hence, there is an urgent demand to enhance the treatment of this malignant cancer.

Anlotinib (AL3818) is a new multi-targeted receptor tyrosine kinase inhibitor (TKI) that is approved for treating advanced non-small cell lung cancer (NSCLC) in patients after more than two series of systematic chemotherapy^5^. It was initially designed to inhibit vascular endothelial growth factor receptor (VEGFR) but is reported to affect other tumour proliferation-related receptor tyrosine kinases, including platelet-derived growth factor α and β, Ret, c-kit, fibroblast growth factor receptor (FGFR) and so forth^6^. Clinical trials of this drug in various cancers, such as sarcoma^7^, renal cell carcinoma^8^ and colorectal tumours^9^, are also under development. Interestingly, compared with previous TKIs (sorafenib and sunitinib), favourable efficacy data for anlotinib in renal cancer patients were reported^8^. This suggests that anlotinib may have alternative antitumour activities than other TKI drugs. To date, anlotinib has been proven to be effective in several types of carcinomas. In synovial sarcoma, anlotinib targets the GINS1 gene and inhibits tumour growth by promoting apoptosis^7^. In hepatocellular carcinoma, anlotinib also induces apoptosis and inhibits proliferation via downregulation of the Erk and Akt pathways^10^. However, the efficacy and mechanism of anlotinib in PDAC is unknown.

The endoplasmic reticulum (ER) is an intracellular organelle that responsible for the three-dimensional folding of newly synthesized proteins. When there is an overload of unfolded or misfolded protein accumulated in the ER lumen, a set of proteins on the ER are activated to re-establish homoeostasis of the ER^11^. The protein overload status in the ER is called ER stress, and the induced adaptive responses are called as the unfolded protein response (UPR), which is involved in a range of cancer treatments. Three pathways are responsible for the UPR, IRE1, ATF6^12,13^ and PERK^14^. The PERK pathway is involved in ROS-induced apoptosis^15^, which is not shared with other pathways. Activation of PERK phosphorylates eukaryotic initiation factor 2 (eIF2α), which then suppresses the overall transcription in the cell but allows the translation of ATF4. ATF4 then triggers apoptosis via activating CHOP, a promotive apoptosis protein that is involved in cell stress and induces cell cycle arrest^16^. In cancer treatment, ER stress is a common process that many antitumour drugs exploit to induce apoptosis in cancer cells, such as bortezomib^17^ and apatinib (a TKI)^18^. Hence, we suspected whether anlotinib has a similar mechanism against PC.

In this study, we demonstrated for the first time that anlotinib was effective for treating PDAC in vivo and in vitro and provided a promising strategy to enhance the efficacy of PDAC treatments.

## Materials and methods

### Cell culture

The three human PC cell lines, PANC-1, BxPC-3 and SW1990, were donated by the Cancer Center of Union Hospital of Tongji Medical College of Huazhong University of Science and Technology (Wuhan, China). PANC-1 cells were maintained in Dulbecco’s modified Eagle’s medium (DMEM, HyClone). BxPC-3 and SW1990 cells were maintained in Roswell Park Memorial Institute 1640 (RPMI-1640, HyClone) medium. The cells were cultured in medium with 10% foetal bovine serum (FBS, Gibco), penicillin (100 U/ml) and streptomycin (100 μg/ml) at 37 °C in a 5% CO_2_ atmosphere.

### Reagents and antibodies

Anlotinib dihydrochloride (AL3818, S8726), and salubrinal (C_21_H_17_Cl_3_N_4_OS, S2923) were purchased from Selleck (Houston, TX, USA). Other reagents: the Cell Counting Kit-8 (CCK-8, CK04) that was purchased from Dojindo (Kumamoto, Japan), the Cell-Light EdU Apollo 643 In Vitro Kit (C10310) form RiboBio (Guangzhou, China), the Annexin V-FITC Apoptosis Detection Kit (APOAF) from Sigma (Sigma Aldrich, St. Louis, MO, USA), the Reactive Oxygen Species Assay Kit (E004-1-1) form Nanjing Jiancheng Bioengineering Institute (Nanjing, China), the Enhanced BCA Protein Assay Kit (P0010) from Beyotime (Shanghai, China), RIPA Buffer (#9806), PMSF (#8553), Phosphatase Inhibitor Cocktail (#5870), from Cell Signalling Technology (CST, Beverly, MA, USA), the PrimeScript RT master mix (RR036A), and the TB Green^®^ Premix Ex Taq™ II (RR820A) from Takara (Shiga, Japan). The antibodies used included cleaved caspase-3 (#9664, 1:1000 dilution), PARP (#9542, 1:1000 dilution), BAX (#5023, 1:1000 dilution), and p-eIF2α (#3398, 1:1000 dilution), which were purchased from Cell Signaling Technology (CST, Beverly, MA, USA). Bcl-2 (12789-1-AP, 1:1000 dilution), PDI (11245-1-AP, 1:200 dilution), PERK (20582-1-AP, 1:1000 dilution), BiP (11587-1-AP, 1:1000 dilution), eIF2α (11170-1-AP, 1:1000 dilution), ATF4 (10835-1-AP, 1:1000 dilution), CHOP (15204-1-AP, 1:1000 dilution), and Ki67 (27309-1-AP, 1:200 dilution) were purchased from Proteintech Group (ProteinTech, Chicago, IL, USA).

### Cell viability assay

A total of 8 × 10^3^ BxPC-3, PANC-1 or SW1990 cells were cultured in each well of the 96-well plates until attachment. Then, 200 μl of complete medium containing 0.1% DMSO or 2.5, 5, 10, 20 or 40 μM anlotinib were added into each of the wells. After 24, 48 or 72 h of treatment, the medium was discarded. Cell viability assay was performed with Cell Counting Kit-8 according to the manufacture’s protocol. The cells were incubated with 100 μl medium containing 10 μl CCK-8 for 4 h. The absorbance was measured by SPECTROstar Nano at 450 nm (BMG LabTech, USA). The inhibition rate (%) was calculated by the following equation: Inhibition rate (%) = 100%−(ODs−ODb)/(ODc−ODb) × 100% (s representing sample, b representing blank, and c representing control). The results and IC50 values that were defined as the concentration of anlotinib resulting in 50% inhibition of cell growth were calculated from concentration-response curves using GraphPad Prism 8.0 (GraphPad Software, Inc., San Diego, CA, USA).

### Colony formation assay

A total of 500 BxPC-3, PANC-1 or SW1990 cells were seeded into 6-well plates and cultured overnight. After discarding the old medium, fresh medium containing 0.1% DMSO or 5, 10 or 20 μM anlotinib was added into each well. After 24 h of treatment, the medium was replaced with 4 ml fresh medium and the PC cells were cultured for 7 days. After fixed with methanol, the cells were washed twice with PBS and stained with 0.05% crystal violet. The colony formation unit was defined as more than 50 cells in a colony. The number of colonies formation unit was then counted and analysed.

### EdU detection

A total of 2.5 × 10^4^ BxPC-3, PANC-1 or SW1990 cells were seeded into each well of the 96-well plates and cultured until attachment. The cells were cultured with 0.1% DMSO or 5, 10 or 20 μM anlotinib for 24 h. EdU detection was performed according to the protocol for the Cell-Light EdU Apollo 643 In Vitro Kit. The cells were cultured with 100 μl medium containing 50 μM EdU for 2 h, followed by washing two times in PBS for 5 min each time. The cells were fixed in 4% paraformaldehyde for 30 min, followed by washing in PBS containing glycine (2 mg/ml). Then, the cells were permeabilized with PBS containing 0.5% Triton X-100 for 10 min, followed by washing in PBS. Then, 100 μl Apollo was added to the cells and cultured for 30 min, followed by washing 3 times in PBS for 5 min each time. Finally, the cells were stained with Hoechst 33342 for 30 min. After staining, the cells were stored at room temperature and protected from the light after washing in PBS. The EdU positive cells were detected by a fluorescence microscope (Life Technologies, ThermoFisher Scientific, USA) analysed by Image-pro-plus 6.0 software (Media Cybernetics, Inc., MD, USA). The results were calculated by the following equation: Percentage of cells (%) = The count of EdU cells/The count of total cells × 100%.

### Cell cycle analysis

The BxPC-3, PANC-1 or SW1990 cells were seeded into 6-well plates and cultured until 70% confluence. Then, the cells were harvested after treatment with different concentration (5, 10 or 20 μM) of anlotinib for 12, 24 or 48 h. The cells were then resuspended in PBS followed by fixation in 75% ice-cold ethanol for overnight. A total of 1 × 10^5^ cells were mixed with 6 μl RNARase (5 mg/ml) at 37 °C for 30 min before adding 13 μl Propidium iodine (PI, 1 mg/ml) at 4 °C in the dark. After 30 min, the cells were then washed and resuspended in PBS (300 μl) for analysis. Then, the data were acquired by a FACSCalibur flow cytometer (Becton Dickinson, USA) applying 488 nm laser excitation, filter: 675/20 for PI and analysed by FlowJo software 10.4 (TreeStar, USA).

### Cell apoptosis analysis

Cell apoptosis was detected following anlotinib treatment by staining using an Annexin V-FITC/PI apoptosis kit. The BxPC-3, PANC-1 or SW1990 cells were harvested after treatment with different concentration (5, 10 or 20 μM) anlotinib for 12, 24 or 48 h and resuspended in binding buffer which contains adequate Ca^+^ according to the protocol of Annexin V-FITC Apoptosis Detection Kit (Sigma, APOAF). A total of 1×10^5^ cells were mixed with 5 μl annexin-V-FITC and 5 μl PI in the dark for 10 min. Then, the data were acquired by a FACSCalibur flow cytometer (Becton Dickinson, USA) applying 488 nm laser excitation, filters: 530/30 for Annexin V-FITC and 675/20 for PI and analysed by FlowJo software 10.4 (TreeStar, USA).

### Reactive oxygen species (ROS) detection

The production of ROS was detected by DCFH-DA diacetate. After treatment with anlotinib, the BxPC-3, PANC-1 or SW1990 cells were cultured with complete medium containing 0.1% DCFH-DA and protected from light at 37 °C for 30 min. A total of 1 × 10^5^ cells were harvested and resuspended in PBS. The cells were then analysed by FACSCalibur flow cytometer (Becton Dickinson, USA) and confirmed by observation under a fluorescence microscope (Life Technologies, ThermoFisher Scientific, USA). The percentage of ROS positive cells was analysed by using FlowJo software 10.4 (TreeStar, USA).

### Immunofluorescence analysis

For immunofluorescence, 2 × 10^4^ BxPC-3, PANC-1 or SW1990 cells were seeded on sterile glass cover slips and placed in 24-well plates, followed by treatment for 24 h. The cells were fixed in 4% paraformaldehyde for 30 min. The cells were then permeabilized with 0.2% Triton X-100 for 15 min. After washing with PBS, the cells were blocked with 5% BSA for 30 min and then incubated with primary antibodies (PDI, 1:200 dilution) at 4 °C overnight. The samples were incubated with secondary antibodies protected from light at 37 °C for 60 min, after washing three times in PBS for 5 min each time. Subsequently, the samples were counterstained with Hoechst 33342 in the dark for 10 min and then observed under a confocal microscope (ZEISS, Germany). The images were analysed by ImageJ software (Rawak Software Inc., Stuttgart, Germany).

### RNA isolation and real-time qPCR

After treatment with anlotinib, cells were harvested and 1 ml TRIzol Reagent was added to each well, followed by the addition of 200 μl chloroform, and incubation at room temperature for 15 min. The cells were centrifuged at 12,000 × *g*, 4 °C for 10 min. The supernatant transferred to a new RNA-free tube and an equal amount of isopropanol was added to the tube. After being gently inverted, the sample was frozen at −20 °C for 30 min and then centrifuged at 12,000 × *g*, 4 °C for 10 min. Subsequently, the sample was washed twice with pre-cooled 75% ethanol, and moderate DEPC water that dissolved total RNA to 500 ng/μl was added for reverse transcription PCR. Real-time qPCR was performed by using TB Green^®^ Premix Ex Taq™ II according to the manufacturer’s protocol (Takara, RR820A). Relative expression of the gene in relation to the housekeeping gene (ACTB) were determined by the comparative Ct method. The mean of Ct values of different groups were used for ΔΔCt and the relative quantification was calculated by the equation RQ = 2^−ΔΔCt^.

### Transfection of siNrf2/shNrf2

The small interfering Nrf2 sequences used were as follows: sense^19^, 5′-GGAGGCAGAUAUGUCUTT-3′; and antisense:5′-AGAUCUAUAUCUUGCCUCCTT-3′. After cells in exponential growth phase were seeded in 6-well plates and reached 50–60 % confluence, cells were transfected with 5 μg siNrf2 or equal molar non-targeting siCtrl by using 10 μl ExFect2000 transfection reagent (Vazyme, China) and incubated at 37 °C in 5% CO_2_. After 42 h, the transfection efficiency of knockdown was identified by the western blot, the siNrf2 or siCtrl cells were used to further experiment. For transfection of shRNA, HEK 293 T cells that were seeded in 60 mm plates and reached 50–60 % confluence, then cultured by Opti-MEM® (Gibco, USA) containing 10 μg pLKO.1-shNrf2 or pLKO.1-scRNA, a nonspecific control RNA, at 37 °C in a 5% CO_2_ atmosphere for harvesting lentiviral particles. When BxPC-3 and PANC-1 cells were reached 50–60 % confluence, medium were replaced with that containing 6 μg/ml polybrene and lentiviral particles of shNrf2 or scRNA. After 72 h, puromycin was added to the medium to select transfected cells. After identified by western blot analysis, the shNrf2 or scRNA cells were harvested to the next analysis.

### Western blot analysis

After treatment with anlotinib, cells were washed in PBS, lysed on ice, and homogenized with RIPA buffer containing PMSF and phosphatase inhibitor for 30 min. After centrifugation at 12,000 × *g* for 30 min, the supernatant was moved to a new tube for analysis of the protein concentrations by an Enhanced BCA Protein Assay Kit (Beyotime, P0010). A total of 30 µg of cellular protein was subjected to 10 or 12% SDS-PAGE and transferred onto a polyvinylidene difluoride membrane (Life Technologies, ThermoFisher Scientific, USA). After incubation with 5% skim milk, the membrane was immunolabeled with primary antibody at 4 °C overnight. The membrane was washed and then incubated with secondary antibody for 2 h at room temperature. After washed by TBST buffer and visualized by horseradish peroxidase substrate (Millipore, Billerica, MA, USA), the signals were detected by chemiluminescence imaging system (GE Healthcare, Piscataway Township, NJ, USA).

### RNA sequencing and bioinformation analysis

RNA sequencing was performed by using Illumina Hiseq2500 platform at Wuhan SeqHealth Tech Co., Ltd. (Wuhan, China). After treatment with anlotinib, cells were homogenized with TRIzol Reagent to extract total RNA. Libraries were constructed and quantified by using Qubit 2.0 (Life Technologies, ThermoFisher Scientific, USA), then the libraries were sequenced on Illumina system for acquiring raw reads. Differentially expressed genes (DEGs) were analysed by cutoff log2 (Fold Change) > 1 and *p* value < 0.05. Biological function analysis of DEGs was enriched by GO and KEGG pathway. The raw data were upload to NCBI Sequence Read Archive and the accession code (PRJNA640938).

### Xenograft studies

Five-week-old female BALB/C nude mice were purchased from Huazhong Agriculture University (Wuhan, China) and approved by Animal Experimental Ethical Inspection of Laboratory Animal Centre (ID Number: HZAUMO-2019–016). All experimental animals were allowed free access to food and water and maintained under specific pathogen-free conditions. The environment was maintained with a 12-h light/dark cycle at 24 ± 2 °C. A total of 100 μl of cellular suspension containing 1×10^7^ PANC-1 or BxPC-3 cells or shNrf2 PANC-1 or BxPC-3 cells were injected subcutaneously into the right hind limbs of the mice. When the tumours grew to ~50 mm^3^, the mice were randomly divided into four groups (*n* = 5) following simple randomization procedures: control group, shNrf2 group, anlotinib group and shNrf2 combined with anlotinib group. Then, 4 mg/kg anlotinib^10,20^ was infused into the mice in the anlotinib group and the co-treatment group by intragastric administration, and an equal volume of PBS was infused into the mice in the other two groups in the same manner.

### Immunohistochemistry

The tumour xenografts were carefully separated from the mice and preserved in 4% paraformaldehyde diluted with 0.1 M PBS at room temperature. The tumour samples were washed and dehydrated by graded ethanol (70–100%), then embedded in paraffin and consecutively sectioned at a thickness of 5 µm. After treated with 3% hydrogen peroxide, the tissue sections were incubated in Tris-EDTA buffer and boiled in a microwave oven for 10 min to complete antigen retrieval. Then the specimens were immersed in 10% goat serum. The slides were incubated with the primary antibody, anti-Ki67 (1:200 dilution), at 4 °C overnight. Then, the sections were washed and marked by a secondary antibody and DAB (BOSTER, China). The results were observed under a microscope (Life Technologies, ThermoFisher Scientific, USA) and analysed by Image-pro-plus 6.0 software. The specimens were evaluated with scores based on staining intensity (0 representing no staining, 1 representing weak staining, 2 representing moderate staining, and 3 representing strong staining) and on the extent of stained cells (0 representing 0%, 1 representing 1–24%, 2 representing 25–49%, 3 representing 50–74%, and 4 representing 75–100%). Then, the total score ranged from 0 to 12 was multiplied with staining intensity and extent of stained cells, and value ≥ 8 points was defined as positive cell. The results were calculated by the following equation: Percentage of Ki67 positive cells (%) = The count of Ki67 positive cells/The count of total cells × 100%.

### Statistical analysis

All samples or animals were assigned randomly and the experiment was performed in a blinded fashion. The size of the sample was calculated considering the power (1-beta) for statistical validity, Difference in Means and Standard Deviation using SigmaPlot 12.0 (Systat Software Inc., San Jose, California, USA). All data were obtained from at least three independent experiments and are presented as the mean ± SD or SEM. The variation estimate of data was obtained and the normal distribution of data was then confirmed. The in vitro data were assessed with one-way analysis of variance (ANOVA). Comparisons in vivo were performed with two-way ANOVA test. The Kaplan–Meier method was used to analyse the overall survival curves. The Bonferroni test was used when significant differences were found *p* < 0.05 was considered significant.

## Results

### Anlotinib inhibits the viability and proliferation of pancreatic cancer cell lines in vitro

**Fig. 1:**
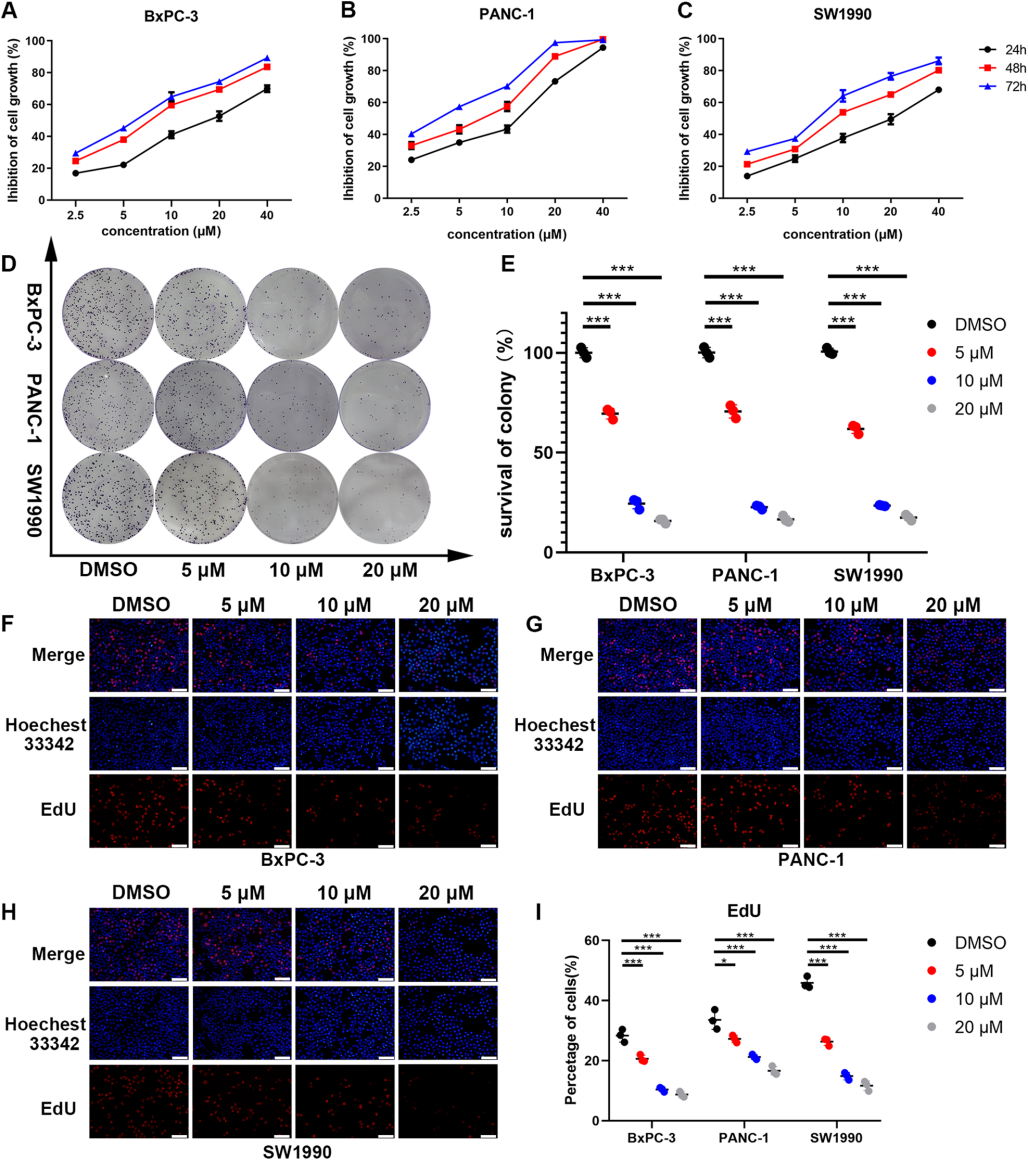
Anlotinib inhibits the proliferation of pancreatic cancer cell lines in vitro. **a**–**c** Dose- and time-dependent curves showing the percentage of inhibition of PC cells. BxPC-3, PANC-1 and SW1990 cells were treated with various concentrations of anlotinib (0–40 μM) for 24, 48 or 72 h. A CCK-8 assay was used to determine the viability of PC cells. **d**–**e** Colony formation was analysed. Each PC cell line was treated with 0, 5, 10 and 20 μM anlotinib for 24 h and cultured for 7 days. **f**–**i** An image (the length of the bar is 100 μm) of EdU staining of PC cells after anlotinib treatment for 24 h. The data are presented as the mean ± SD from three independent experiments (*p < 0.05; **p < 0.01; ***p < 0.001).

To evaluate the effect of anlotinib on the proliferation of PC cell lines. The three PC cell lines were treated with anlotinib at increasing concentrations (0, 2.5, 5, 10, 20 or 40 μM) for 24, 48 or 72 h. A CCK-8 assay was then carried out to evaluate the viability of the cells. The inhibition of anlotinib on each cell line exhibited dose- and time-dependent effects (Fig. 1a–c). The IC50 of the cell lines ranged from 3.762 to 18.31 μM at different concentrations and durations of treatment (Table 1). The morphological changes in PC cells cultured with increasing concentrations of anlotinib were observed under a microscope. Cells lost their normal morphology and became round and detached from the plate (Supplementary file: Fig. S1). The colony formation assay was used to validate the anti-proliferative effects of anlotinib on PC cells. After treatment with anlotinib for 24 h at concentrations of 0, 5, 10 and 20 μM, the cells were cultured with fresh complete medium for 7 days, and the number of colonies was counted. The number of colonies decreased as the concentration of anlotinib increased (Fig. 1d, e). In addition, the EdU detection assay showed similar results as the colony formation assay. EdU is the terminal alkaline-containing nucleoside analogue of thymidine and is incorporated into DNA during active DNA synthesis. The intensity of EdU in the cells indicates the number of cells undergoing active proliferation and that are in S phase. In this study, the EdU detection assay at 24 h showed that the percentage of labelled cells decreased in the PANC-1, BxPC-3 and SW1990 cell lines as the concentration of anlotinib increased (Fig. 1f-i). These results confirmed that anlotinib elicits anti-proliferative effects on various PC cell lines in vitro, which depends on the dose and duration of the treatment.

### Anlotinib induces G2/M phase arrest and apoptosis in PC cell lines

**Fig. 2:**
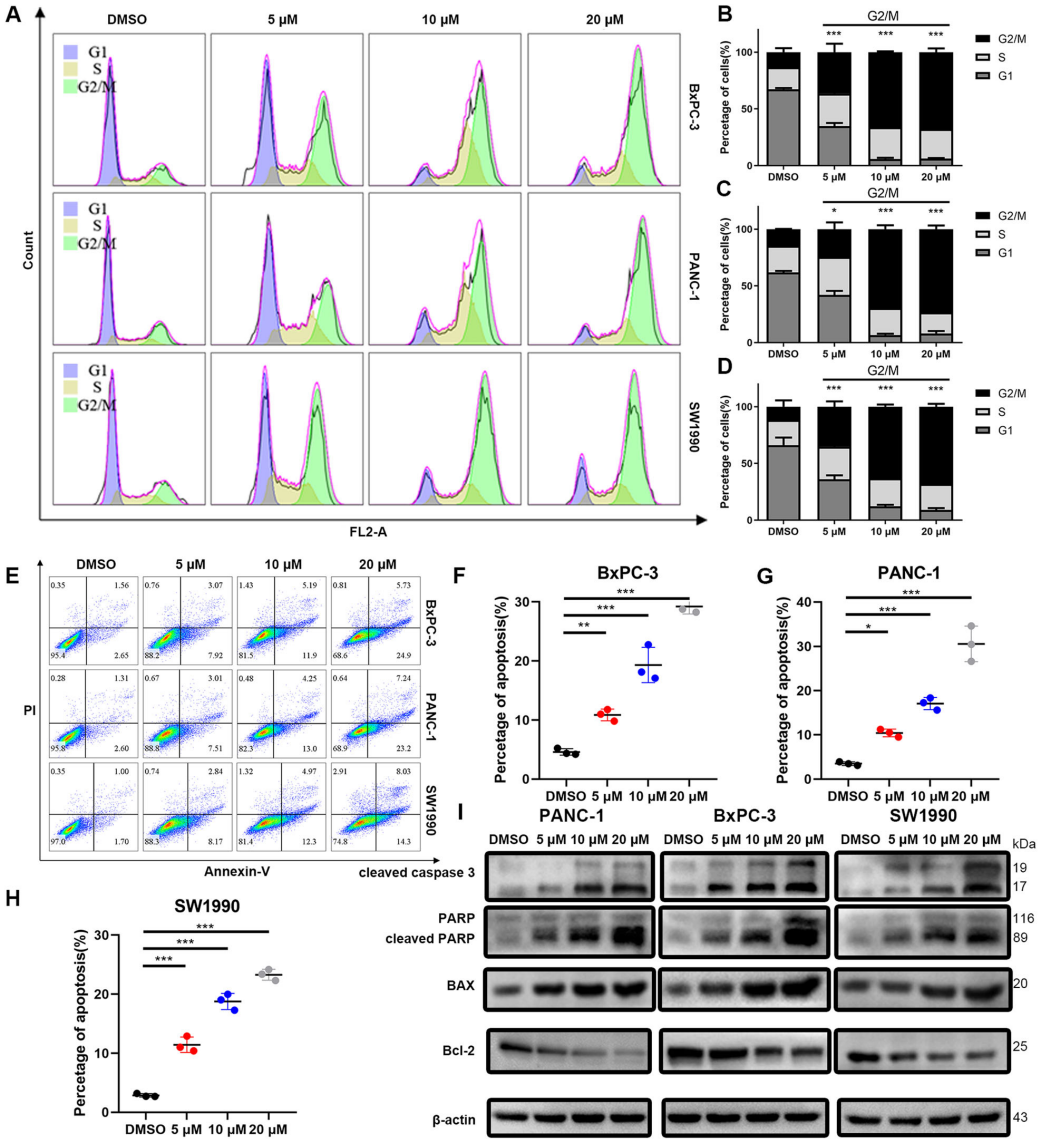
Anlotinib induces G2/M phase arrest and apoptosis. **a**–**d** Anlotinib-induced G2/M phase arrest. The cells were treated with anlotinib for 24 h and analysed by flow cytometry after staining with PI. **e**–**h** After treatment with anlotinib, the cells were stained with PI and Annexin-V-FITC to detect apoptosis, followed by flow cytometry. The population of cells in the lower left quadrant shows the live cells (Annexin V-/PI-), population in lower right quadrant shows the early apoptotic cells and that in upper right quadrant shows the late apoptotic or dead cells. Cells in state of early apoptosis, late apoptosis and death cells were counted in apoptotic cells. **i** Western blotting experiments detected several markers that reflect the apoptosis level of cells. The data are presented as the mean ± SD from 3 independent experiments (*p < 0.05; **p < 0.01; ***p < 0.001).

To investigate the mechanism of the anti-proliferative effects of anlotinib on PC, the distribution of the cell cycle was evaluated. The cell lines were treated with various concentrations of anlotinib for 24 h and analysed by flow cytometry after staining with PI. As shown in Fig. 2a–d, the fraction of G2/M phase cells increased, and the fraction of G1 cells decreased in a dose-dependent manner. The fraction of G2/M phase cells treated with 10 μM anlotinib increased for 24 or 48 h, but did not significantly increase for 12 h (Supplementary file: Fig. S3A–D). In addition, the PC cells were treated with anlotinib (0, 5, 10 and 20 μM) for 12, 24 or 48 h and stained by Annexin-V-FITC and PI, followed by flow cytometry analysis. The percentage of apoptotic cells increased in a dose-dependent manner (Fig. 2e–h and Supplementary file: Fig. S3E–H). Consistently, the expression of cleaved caspase 3, cleaved PARP and BAX were upregulated, and the expression of Bcl-2 was downregulated in a dose-dependent manner (Fig. 2i). Meanwhile, the expression of apoptotic proteins increased in a time-dependent manner (Supplementary file: Fig. S3I). These results demonstrated that anlotinib-induced apoptosis in PC cells.

### Gene expression profiles identified anlotinib-affected pathways in PC cells

To determine the mechanism by which anlotinib affects PC cells, we compared the expression profiles of genes in the BxPC-3 cell line, from the primary tumour of adenocarcinoma, treated with anlotinib and the control group using high-throughput sequencing. We found 190 upregulated genes and 146 downregulated genes in the transcriptome data. Differences in expression levels were defined by cutoff log2 (fold change) > 1 (Supplementary file: Fig. S2A). GO enrichment analysis revealed that most of the differentially expressed genes are associated with apoptosis, ER stress, and oxidation-reduction processes (Supplementary file: Fig. S2B–D). The proteins associated with ER stress are shown in a heatmap (Supplementary file: Fig. S2E). These results highlight that anlotinib affects apoptosis, ER stress and oxidation-reduction processes in PC cells.

### Anlotinib-induced apoptosis via ER stress

**Fig. 3:**
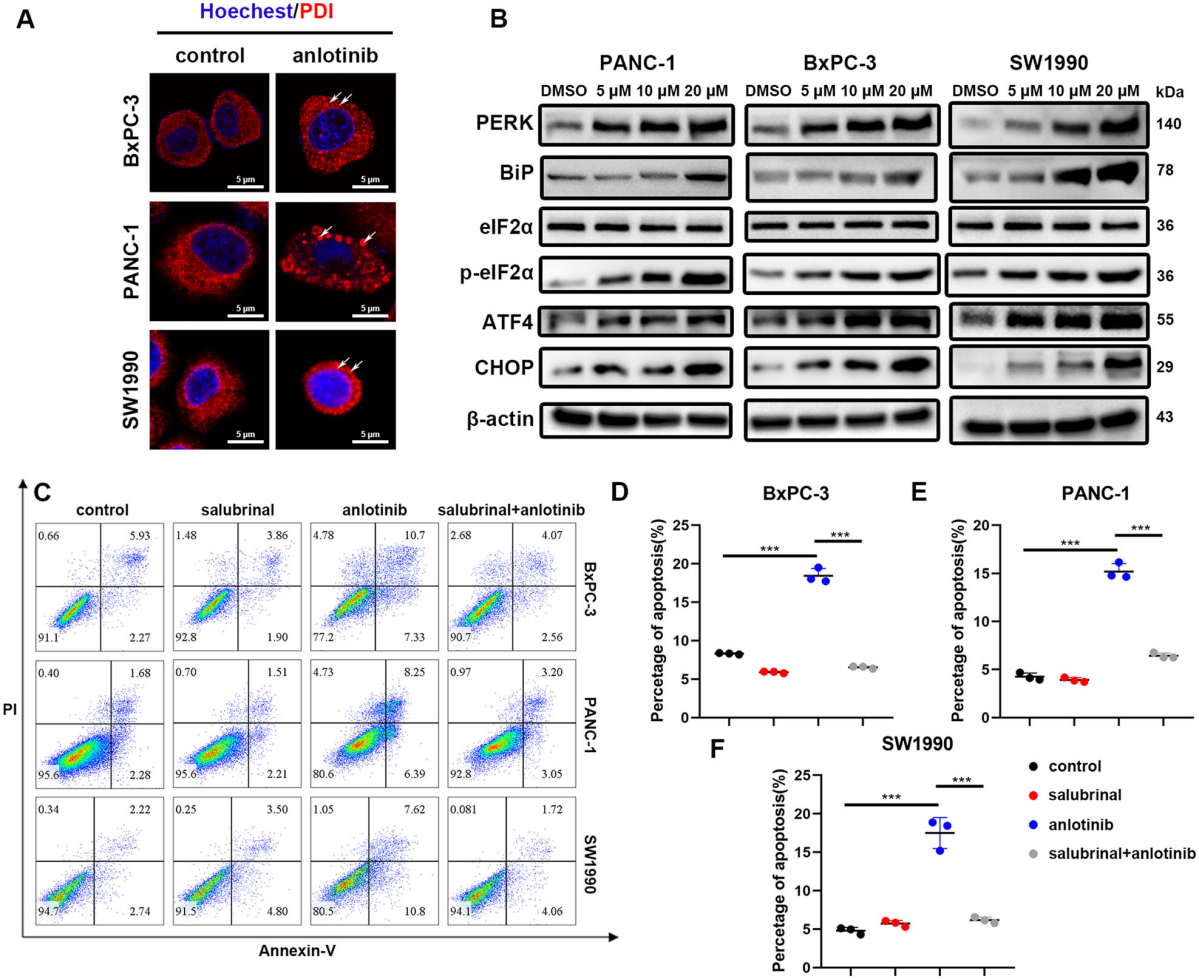
Anlotinib leads to apoptosis via endoplasmic reticulum stress. **a** Immunofluorescent analysis was performed to detect the ER luminal marker protein disulphide isomerase (PDI). **b** The expression of the indicated markers reflecting endoplasmic reticulum stress in three PC cell lines was examined by western blotting. **c**–**f** PC cell apoptosis was detected using PI and Annexin-V-FITC staining. The data are presented as the mean ± SD from three independent experiments (***p < 0.001).

ER stress is a common pathway through which chemotherapy drugs induce apoptosis in cancer cells. ER stress activates apoptosis pathways and then causes cell apoptosis. In this study, the mRNA transcriptome showed that the genes related to ER stress were differentially expressed in the anlotinib-treated group; thus, we hypothesized that ER stress participates in anlotinib-induced apoptosis in PC cells. To test this hypothesis, the ER luminal marker protein disulphide isomerase (PDI) was then detected by immunostaining in anlotinib-treated PC cells. The striking aggregation of PDI, which is a hallmark of ER stress, was also observed under a confocal microscope when the PC cell lines were treated with anlotinib for 24 h (Fig. 3a). Proteins in the ER stress pathway, including PERK, BiP, ATF4, CHOP, eIF2α, and p-eIF2α, were also increased in the anlotinib-treated group in a dose-dependent manner (Fig. 3b). To further investigate the causal relationship between ER stress and apoptosis, PC cells treated with anlotinib were cultured with or without salubrinal, an inhibitor of eIF2α phosphorylation^21^. The flow cytometry results showed that the percentage of apoptotic cells was markedly decreased by anlotinib combined with salubrinal compared with that of treatment with anlotinib alone (Fig. 3c–f). These results confirmed that anlotinib induces apoptosis in PC cell lines via ER stress.

### Reactive oxygen species are critical in ER stress-activated apoptosis

**Fig. 4:**
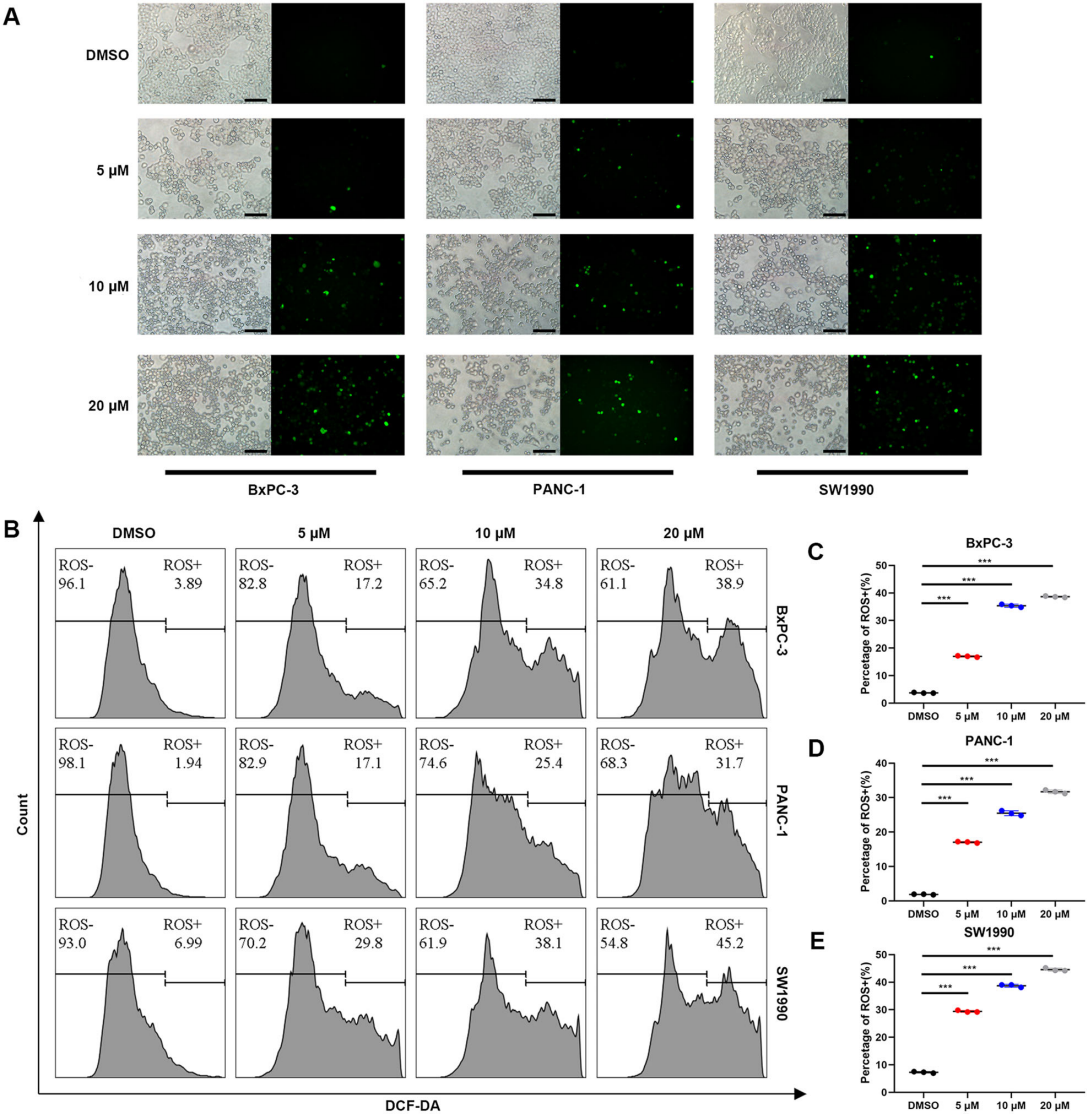
ROS are critical in endoplasmic reticulum stress-induced apoptosis. **a** After the PC cells were treated with anlotinib, the cells were stained with DCFH-DA, followed by observation under a fluorescence microscope (the length of the bar is 100 μm). **b**–**e** After treatment and staining, the ROS levels in cells were detected by flow cytometry (mean ± SD, ***p < 0.001).

ROS are a major contributor to ER stress. We, therefore, hypothesized that ROS are involved in anlotinib-induced ER stress. To confirm this hypothesis, PC cells treated with anlotinib were stained with DCFH-DA. As shown in Fig. 4a, anlotinib increased the percentage of cells with fluorescence in a dose-dependent manner after adding anlotinib. The same result was found by flow cytometry in Fig. 4b–e. These results revealed that anlotinib is associated with the production of ROS in a dose-dependent manner.

**Fig. 5:**
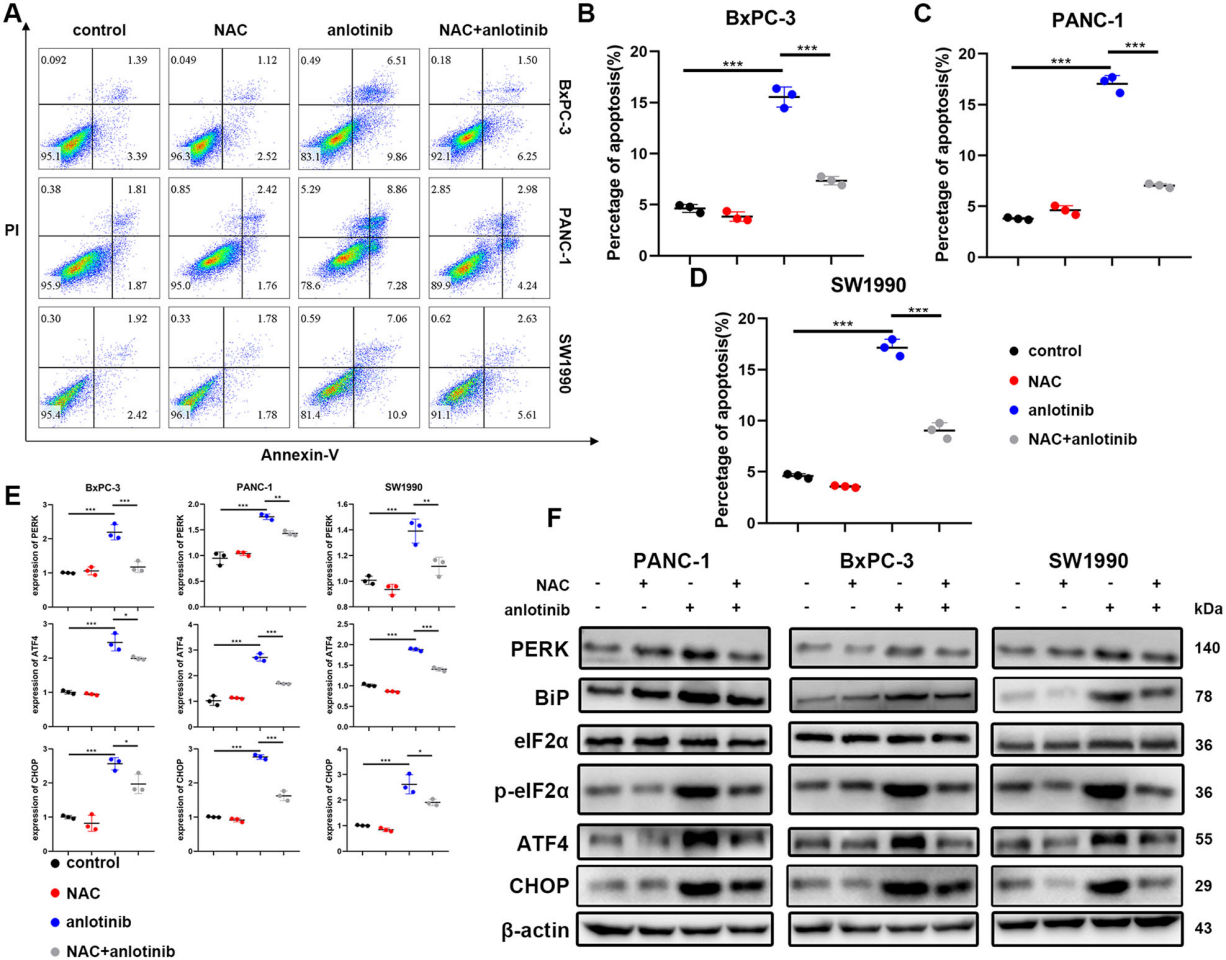
The inhibition of ROS reduces endoplasmic reticulum stress and apoptosis. **a**–**d** After the PC cells were treated with or without anlotinib or NAC, the cells were stained with PI and Annexin-V-FITC and detected by flow cytometry (***p < 0.001). **e** The mRNA expression of genes in the unfolded protein response was detected by real-time qPCR, and the relative quantification was calculated by the equation RQ = 2^−ΔΔCt^ (mean ± SD, *p < 0.05; **p < 0.01; ***p < 0.001). **f** The cells were detected using the UPR marker protein after treatment with or without anlotinib or NAC.

Furthermore, PC cells treated with anlotinib were cultured with or without N-acetyl-cysteine (NAC), an ROS scavenger. The percentage of apoptotic cells was markedly decreased by NAC combined with anlotinib compared to that of treatment with anlotinib alone (Fig. 5a–d). We then investigated whether ER stress is involved in ROS-induced apoptosis in anlotinib-treated PC cells. As expected, the mRNA expression of BiP, ATF4 and CHOP (Fig. 5e) increased in the group treated with anlotinib compared with that of the control group and decreased after anlotinib treatment combined with NAC. Likewise, the protein expression of BiP, PERK, p-eIF2α, and CHOP was downregulated in the group treated with anlotinib combined with NAC compared with that of the group treated with anlotinib alone (Fig. 5f). These results demonstrated that ER stress is induced by ROS production, which mediates anlotinib-induced apoptosis in PC cell lines.

### Suppression of Nrf2 enhanced anlotinib-induced apoptosis

**Fig. 6:**
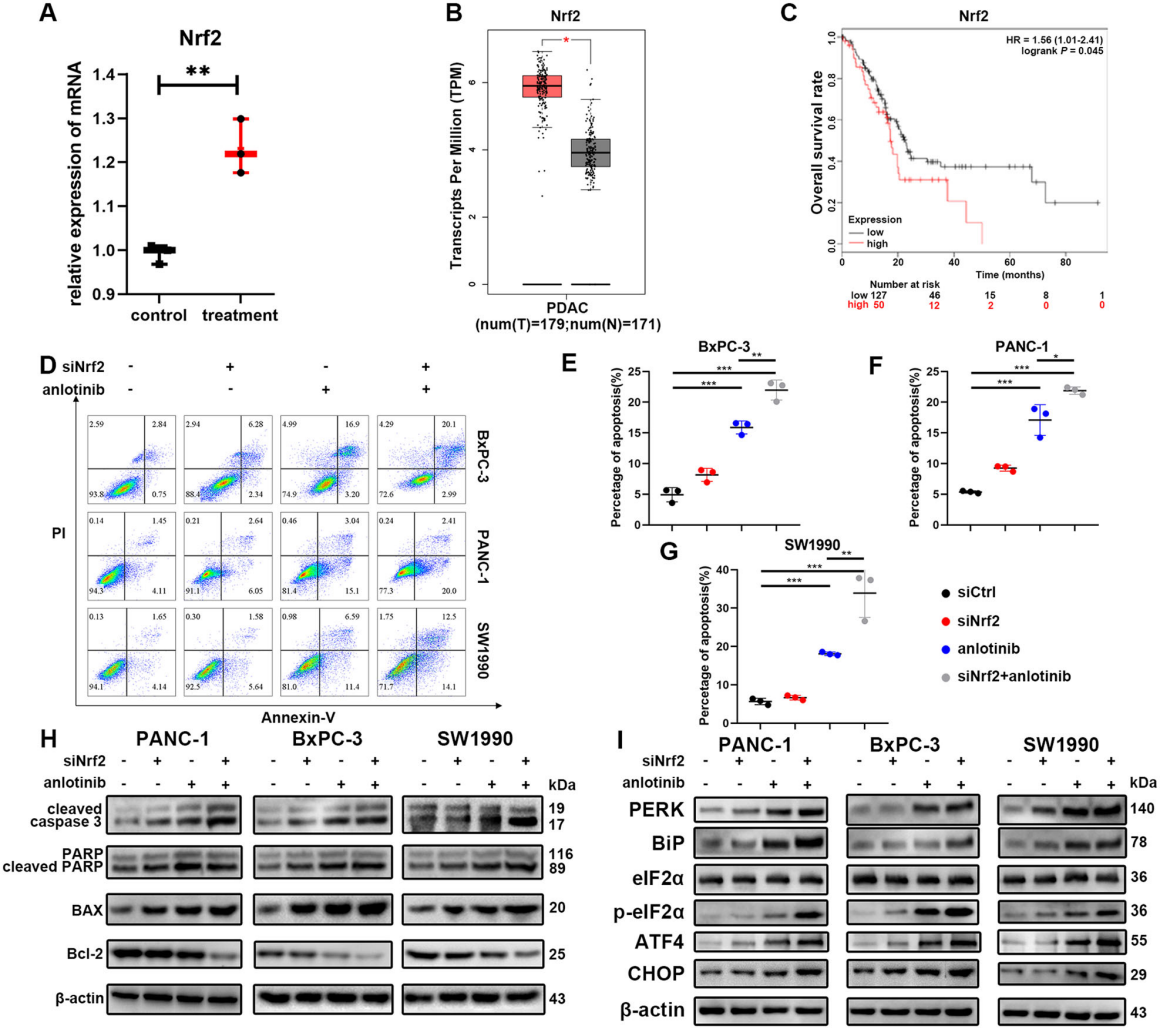
Suppression of Nrf2 enhances the antitumour effect of anlotinib in vitro*.* **a** Differentially expressed Nrf2 was determined using high-throughput sequencing (mean ± SD, **p < 0.01). **b** The relationship between the expression of Nrf2 transcripts in pancreatic cancer patients. **c** A K-M analysis indicated that high expression of Nrf2 was associated with pancreatic cancer patients (*p* = 0.045). **d**–**g** After PC cells were treated with or without anlotinib or siNrf2, the cells were stained with PI and Annexin-V-FITC and detected by flow cytometry (mean ± SD, *p < 0.05; **p < 0.01; ***p < 0.001). **h** After PC cells were treated with or without anlotinib or siNrf2, several markers reflecting the apoptosis level were detected by western blotting. **i** Several unfolded protein response markers were detected by western blotting.

Our work suggested that ROS production was important in anlotinib-induced apoptosis in PC. There are balanced mechanisms to protect cells from ROS injury, and increased Nrf2 is one of those mechanisms. Nrf2, an antioxidant factor that regulates numerous genes to neutralize free radicals, was also significantly increased in the anlotinib-treated group (Fig. 6a). Nrf2 has been proven to be a protective protein in some cancers, but its role in PC is unknown. Compared with expression in normal samples, PC tumours have higher Nrf2 expression of transcripts (http://gepia.cancer-pku.c/) (Fig. 6b). A Kaplan–Meier (K-M) analysis indicated that Nrf2 expression was associated with poor prognosis in patients with PC. Analysis of the Nrf2 gene in 177 patients with PC showed that the overall survival rate of patients with low Nrf2 gene expression is better than that of patients with high Nrf2 gene expression (http://kmplot.com/analysis/index.php?p=service) (Fig. 6c). Therefore, we hypothesized that Nrf2 promotes the progression of PC by protecting tumour cells from anlotinib-induced oxidative injury. To verify this hypothesis, anlotinib-induced apoptosis was examined in siNrf2 PC cells and the treated control group by flow cytometry. We found that the percentage of apoptotic cells in the anlotinib combined with siNrf2 group was higher than that of the anlotinib group (Fig. 6d–g). The siNrf2 cell lines were identified by western blotting (Supplementary file: Fig. S4A). The expression of cleaved caspase 3, cleaved PARP and BAX was upregulated, and the expression of Bcl-2 was downregulated in the co-treatment group compared with that of anlotinib alone (Fig. 6h). Proteins in the ER stress pathway, including PERK, BiP, ATF4, CHOP, eIF2α, and p-eIF2α, were also increased in the co-treatment group (Fig. 6i). The results showed that the suppression of Nrf2 combined with anlotinib significantly promoted the apoptosis of PC cells compared with that of anlotinib alone.

### Tumour suppression induced by anlotinib is enhanced by suppression of Nrf2

**Fig. 7:**
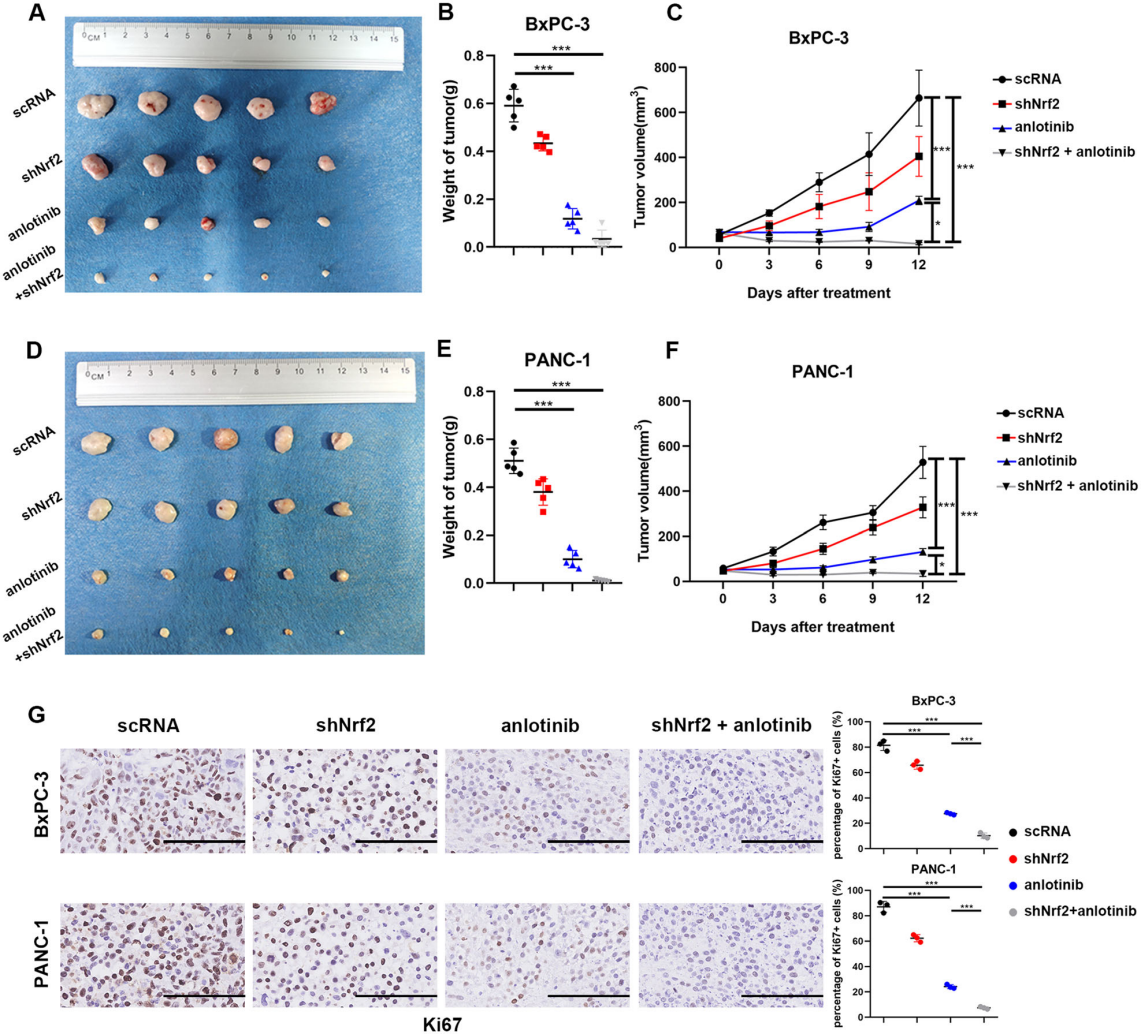
Tumour suppression induced by anlotinib is enhanced by suppression of Nrf2 in vivo. **a** Images of BxPC-3 cells or shNrf2 BxPC-3 cells xenograft tumours at the end of the treatment. BALB/C mice (*n* = 5) were injected subcutaneously with 1 × 10^7^ BxPC-3 cells. These mice were sacrificed after 12 days of treatment with or without anlotinib (4 mg/kg), and the tumours were dissected and photographed. **b** The weights of the BxPC-3 cell xenograft tumours at the end of the treatment (***p < 0.001). **c** Tumour volumes in BALB/C mice during the 12-day treatment (mean ± SEM, *p = 0.0339, ***p < 0.001). **d**–**f** (**d**) Image, (**e**) weight and (**f**) volume (mean ± SEM, *, *p* = 0.0389, ***, *p* < 0.001) of PANC-1 or shNrf2 PANC-1 cell xenograft tumours. **g** After treatment as indicated above, Ki-67 protein expression was detected in sections of xenograft tumours by immunohistochemical analysis (the length of the bar is 100 μm).

Similar to the in vitro results, Nrf2 suppression combined with anlotinib treatment was better than treatment with anlotinib alone. The shNrf2 cell lines were identified by western blotting (Supplementary file: Fig. S4B). A total of 1 × 10^7^ BxPC-3 or PANC-1 cells were subcutaneously injected into right hind limbs of BALB/C nude mice. After the tumour volumes grew ~50 mm^3^, the mice were treated with anlotinib (4 mg/kg) for 12 days. Significantly, the tumour volumes of the shNrf2 combined with anlotinib group were smaller than those of the anlotinib alone group. The tumour weights showed a similar trend as the results of tumour volumes (Fig. 7a–f). Moreover, immunochemistry results showed that Ki67, a biomarker of proliferation, had the lowest signal in the group treated with anlotinib combined with shNrf2 compared to that of other groups (Fig. 7g). In summary, the suppression of Nrf2 significantly promoted the apoptotic effect of anlotinib in human PC cells in vivo.

**Table 1:** The Half maximal inhibitory concentration (IC50) of three PC cell lines.

IC50 (μmol/L)		Time		
		24 h	48 h	72 h
Cell lines				
BxPC-3		16.36	7.568	5.616
PANC-1		8.922	5.604	3.762
SW1990		18.31	9.894	6.881

## Discussion

PC is one of the leading causes of cancer-related deaths worldwide. Until now, the mainstream medical treatment option for patients with PC has not been desirable^22^. Therefore, our work may provide a new therapeutic strategy for human PC.

As an anti-angiogenic drug selectively targeted to VEGFR2, anlotinib has been shown to have broad-spectrum antitumour activity in patients with metastatic soft-tissue sarcoma^23^ and thyroid cancer^24^ mainly through its anti-angiogenesis effect. However, our experiments suggested that anlotinib directly inhibits PC cells rather than inhibits angiogenesis. We suspected that an unknown mechanism other than anti-angiogenesis was responsible for its antitumour effects. Anlotinib inhibits proliferation and promotes apoptosis by directly affecting the ratio of Bcl-2/BAX in hepatocellular carcinoma^10^. Likewise, in this study, we also found that the anti-apoptotic factor Bcl-2 was downregulated and that the pro-apoptotic factor BAX was upregulated, as well as cleaved caspase 3 and cleaved PARP, which are involved in the activation cascade of caspases responsible for apoptosis. Therefore, we believe that anlotinib directly causes apoptosis of PC cells.

Cancer cells are characterized by elevated aerobic glycolysis and high levels of oxidative stress^25^. This increase in oxidative stress is usually due to the accumulation of ROS. The tumour cells have higher ROS levels than normal cells and have a balanced increased antioxidant mechanism^26^. However, excessive levels of ROS still cause cytotoxicity via regulation of DNA damage, autophagy, metabolism, migration and ER stress^27–29^. We found that increased ROS by anlotinib in PC cells would induce ER stress. This was also supported by our high-throughput sequencing data, in which oxidative stress-related genes were activated in anlotinib-treated cells. ROS-induced ER stress is a mechanism utilized by other chemotherapeutic drugs to induce apoptosis in tumour cells^30^. A study reported that sorafenib, another TKI, causes ROS elevation by mediating the production of O_2_^•−^, a superoxide free radical that is a major cause of ROS, in hepatocellular carcinoma^31^. We postulated that when the oxidation-reduction system was disrupted under anlotinib in our study, the oxygen was then incompletely reduced to O_2_^•−^, causing the accumulation of ROS. Therefore, we used N-acetylcysteine (NAC) to scavenge ROS and found that apoptosis of PC cells and the level of proteins related to ER stress decreased, suggesting that ROS are upstream signals of ER stress.

**Fig. 8:**
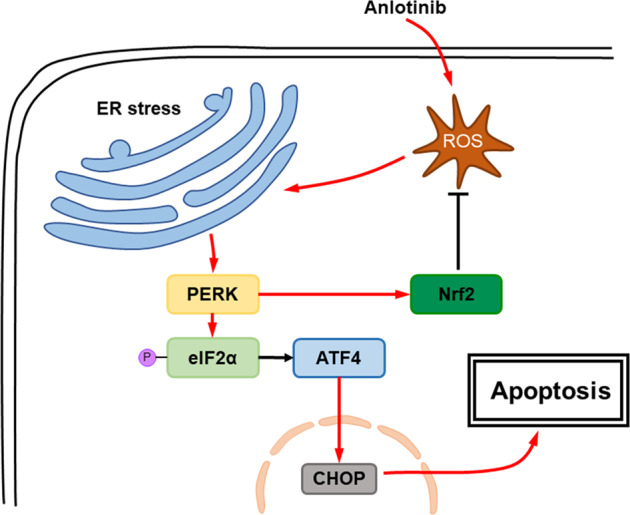
Schematic diagram of anlotinib-induced apoptosis in PC cells. Anlotinib increases ROS, causing endoplasmic reticulum stress. Thus, apoptosis-related proteins promote apoptosis of the cells. Moreover, PERK activates Nrf2, which inhibits the production of ROS.

The ER is an important organelle that maintains cell homeostasis to ensure the normal synthesis of proteins. When cells are subjected to strong external environmental stress, the unfolded or misfolded proteins accumulate in the ER, causing ER stress. Many drugs, such as bortezomib^17^, suffruticosa aqueous extracts^32^ and apatinib^18^, cause ER stress and then induce apoptosis. Similar to these reports, our study also found that anlotinib induced increases in BiP, PERK and CHOP, which are ER stress biomarker proteins. Elevated BiP and PERK are reported to activate downstream eIF2α and to increase its phosphorylation level in response to treatment with anlotinib. The upregulation of p-eIF2α then activates downstream ATF4, which promotes the increase in the pro-apoptotic protein CHOP and ultimately causes apoptosis (Fig. 8). As a critical transcription factor for ER-induced apoptosis, CHOP promotes the transcription of the ER stress proteins GADD34 and ERO1 and inhibits transcription of the anti-apoptotic molecule Bcl2, thereby promoting apoptosis. In this study, we cultured PC cells treated with anlotinib and salubrinal, an inhibitor of eIF2α phosphorylation, and found that the anlotinib-induced apoptosis was significantly reduced, indicating that ER stress is the pivotal process involved in the apoptosis of anlotinib-treated PC. Therefore, we demonstrated that anlotinib-induced ROS damage the ER membrane or other membrane structure, which cause ER stress. The ER stress then further promote the apoptosis in PC cells.

Oxidative stress and antioxidant agents are important in various cancers. Along with the chemotherapeutic drug-induced accumulation of ROS in PC cells, a series of adaptive changes are made to balance cell homeostasis. To handle the high level of ROS, tumour cells have a corresponding antioxidant mechanism^33,34^. Antioxidant treatment or activation of endogenous antioxidants such as Nrf2 reduces ROS and promotes early lung tumour progression^35^. A study also showed that knockdown of the Nrf2 gene enhanced the chemosensitivity of biliary tract cancer xenograft models in mice^36^. As an antioxidant protein, Nrf2 achieves its functions by regulating downstream detoxification factors, eliminating oxidized proteins and neutralizing electrophilic agents, thereby enhancing cellular antioxidant capacity. It has been reported that Nrf2 is activated by PERK in studies of multidrug resistance^37^. Since we found that both PERK and Nrf2 were upregulated with anlotinib treatment and the ROS production is important in anlotinib-induced apoptosis, we, therefore, considered that the increased expression of Nrf2 in anlotinib-treated PC cells would protect tumours from ROS injury and decreases the tumouricidal effect. To eliminate the adverse effect of Nrf2 in PC cells with anlotinib treatment, we then used shNrf2 combined with anlotinib to treat PC. The results showed that suppression of Nrf2 enhanced the efficacy with anlotinib treatment, significantly inhibiting the volumes of subcutaneous tumours in a xenograft model. Therefore, suppression of Nrf2 expression increased the drug sensitivity to anlotinib in PC cells.

In conclusion, our work found that anlotinib showed tumouricidal effects in PC cells both in vivo and in vitro. ROS-induced ER stress is a novel mechanism by which anlotinib causes apoptosis in PC cells. Furthermore, we found that suppressing Nrf2 significantly improved the tumouricidal effectiveness of anlotinib for PC cells Hence, our study provides a theoretical basis for the application of anlotinib in PC. The combination of anlotinib with an Nrf2 inhibitor may provide a new strategy for the treatment of PC in the future.

## Supplementary information

Supplementary figure legends

Supplementary figure 1

Supplementary figure 2

Supplementary figure 3

Supplementary figure 4
