# Supplementary material for: Reactive oxygen species mediate anlotinib-induced apoptosis via activation of endoplasmic reticulum stress in pancreatic cancer
Source: Cell Death Dis. 2020 Sep 17;11(9):766. doi: 10.1038/s41419-020-02938-4 (PMC7499216; doi:10.1038/s41419-020-02938-4)
Supplement: Supplementary file 1 — Supplementary figure legends [file 41419_2020_2938_MOESM1_ESM.docx]

**Supplemental Figure 1.** The morphological changes in PC cells cultured with increasing concentrations of anlotinib were observed under a microscope. The length of the bar is 100 μm.

**Supplemental Figure 2.** Differentially expressed genes (DEGs) using high-throughput sequencing. (A) The volcano plot of the DEGs. The number of DEGs was accounted for, and the indicated colour is the same as above. The X-axis represents log2 (fold change), and the Y-axis represents -log10 (*p-value*). (B-D) GO enrichment analysis of DEGs; *p-value* < 0.01. The X-axis represents the number of genes, the Y-axis represents the name of biological process or cellular compound and the orange bar represents biological process, and the green bar represents the cellular compound. (E) Hierarchical clustering of differentially expressed genes in the endoplasmic reticulum unfolded protein response (by using cutoff log2 (FC)>1 and *p value*<0.05). Analysis was performed with log10 (RPKM) values, with red indicating a highly expressed gene and blue indicating a low expressed gene. The X-axis represents the different samples, and the Y-axis represents the gene name.

**Supplemental Figure 3.** Anlotinib induces G2/M phase arrest and apoptosis in different time. (A-D) Anlotinib induced G2/M phase arrest. The cells were treated with anlotinib for 12 h to 48 h and analysed by flow cytometry after staining with PI. (E-H) The apoptotic rate of PC cells treated with anlotinib in different time (0, 12, 24 and 48 h). The population of cells in the lower left quadrant shows the live cells (Annexin V-/PI-), population in lower right quadrant shows the early apoptotic cells and that in upper right quadrant shows the late apoptotic or dead cells. Cells in state of early apoptosis, late apoptosis and death cells were counted in apoptotic cells. (I) Western blotting experiments detected several markers that reflect the apoptosis level of cells in different time. The data are presented as the mean ± SD from 3 independent experiments (*, p < 0.05; **, p < 0.01; ***, p < 0.001).

**Supplemental Figure 4.** Identification of siNrf2 and shNrf2. (A) The western blotting of siRNA and siCtrl. (B) The western blotting of shRNA and scRNA.
