# Supplementary figures and images for: Reactive oxygen species mediate anlotinib-induced apoptosis via activation of endoplasmic reticulum stress in pancreatic cancer
Source: Cell Death Dis. 2020 Sep 17;11(9):766. doi: 10.1038/s41419-020-02938-4 (PMC7499216; doi:10.1038/s41419-020-02938-4)

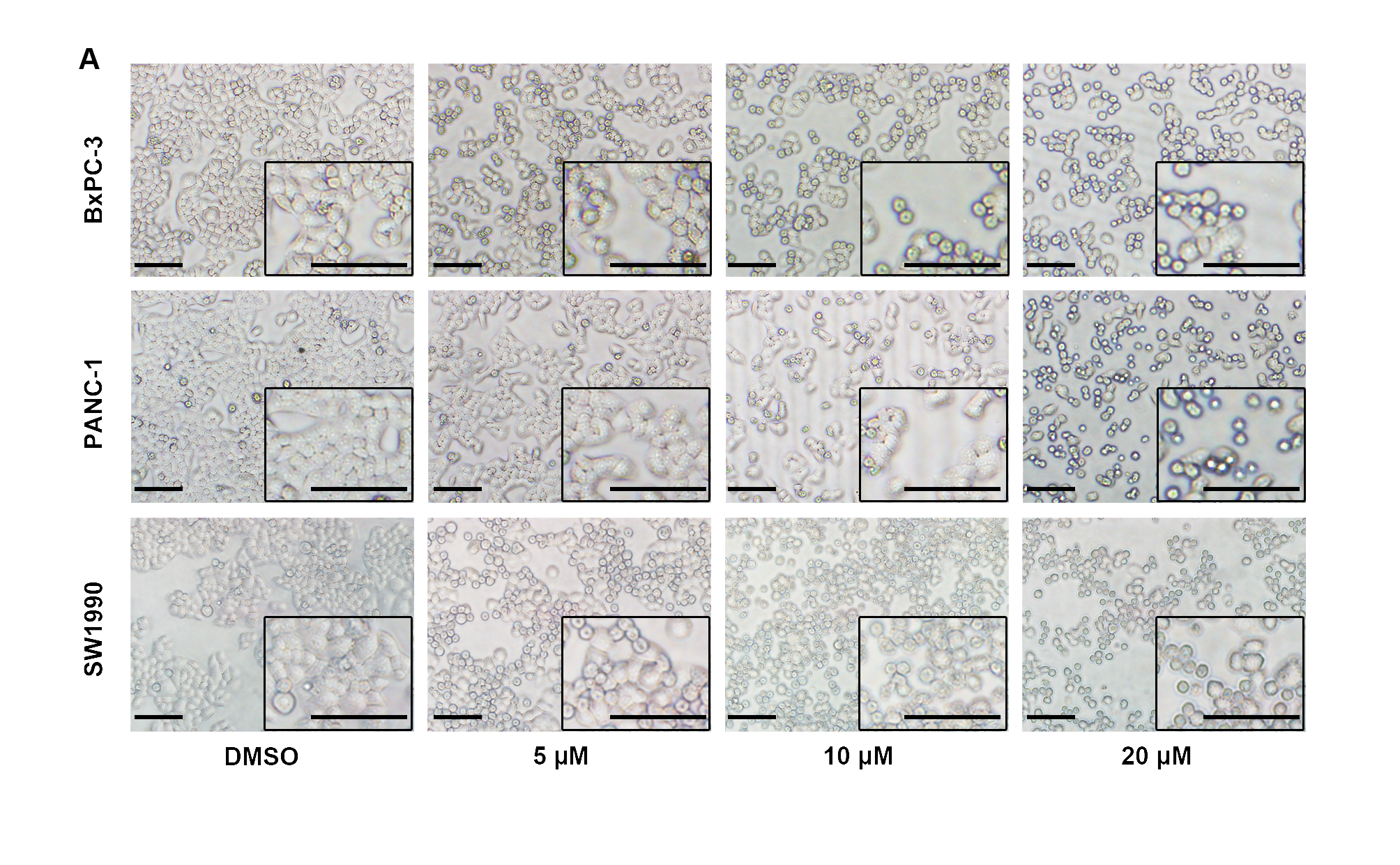

Supplement: Supplementary file 2 — Supplementary figure 1 [file 41419_2020_2938_MOESM2_ESM.tif]

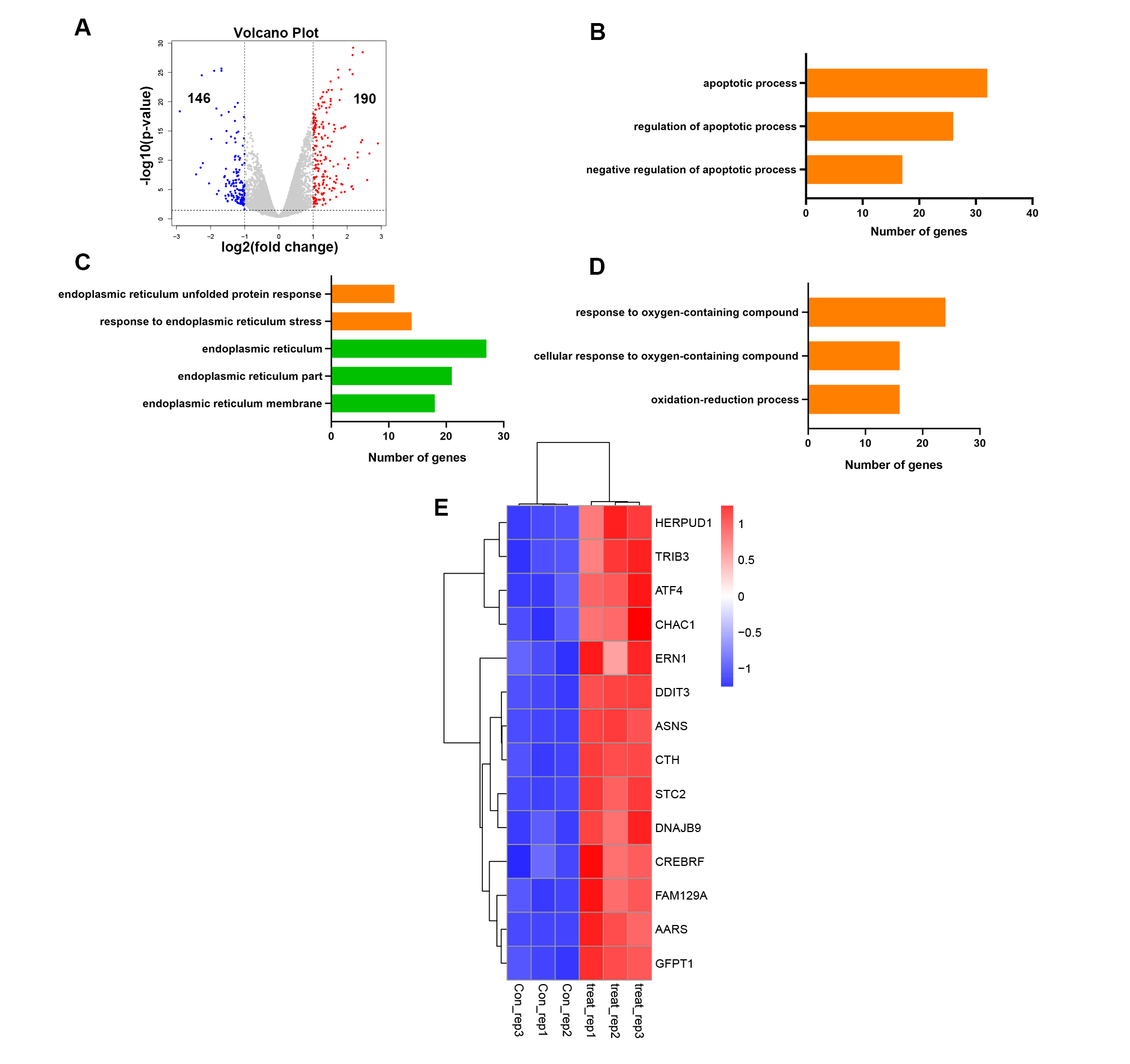

Supplement: Supplementary file 3 — Supplementary figure 2 [file 41419_2020_2938_MOESM3_ESM.tif]

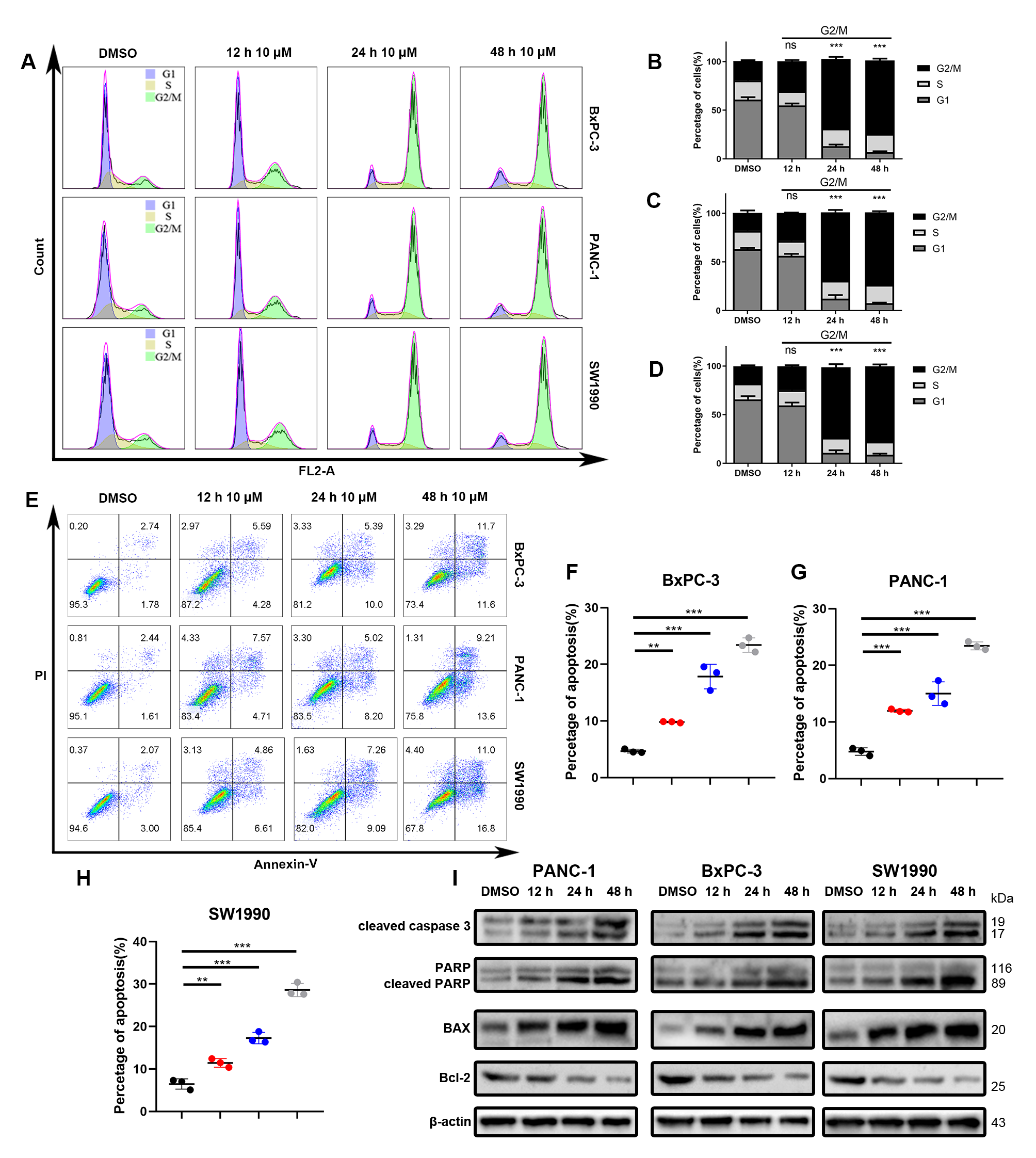

Supplement: Supplementary file 4 — Supplementary figure 3 [file 41419_2020_2938_MOESM4_ESM.tif]

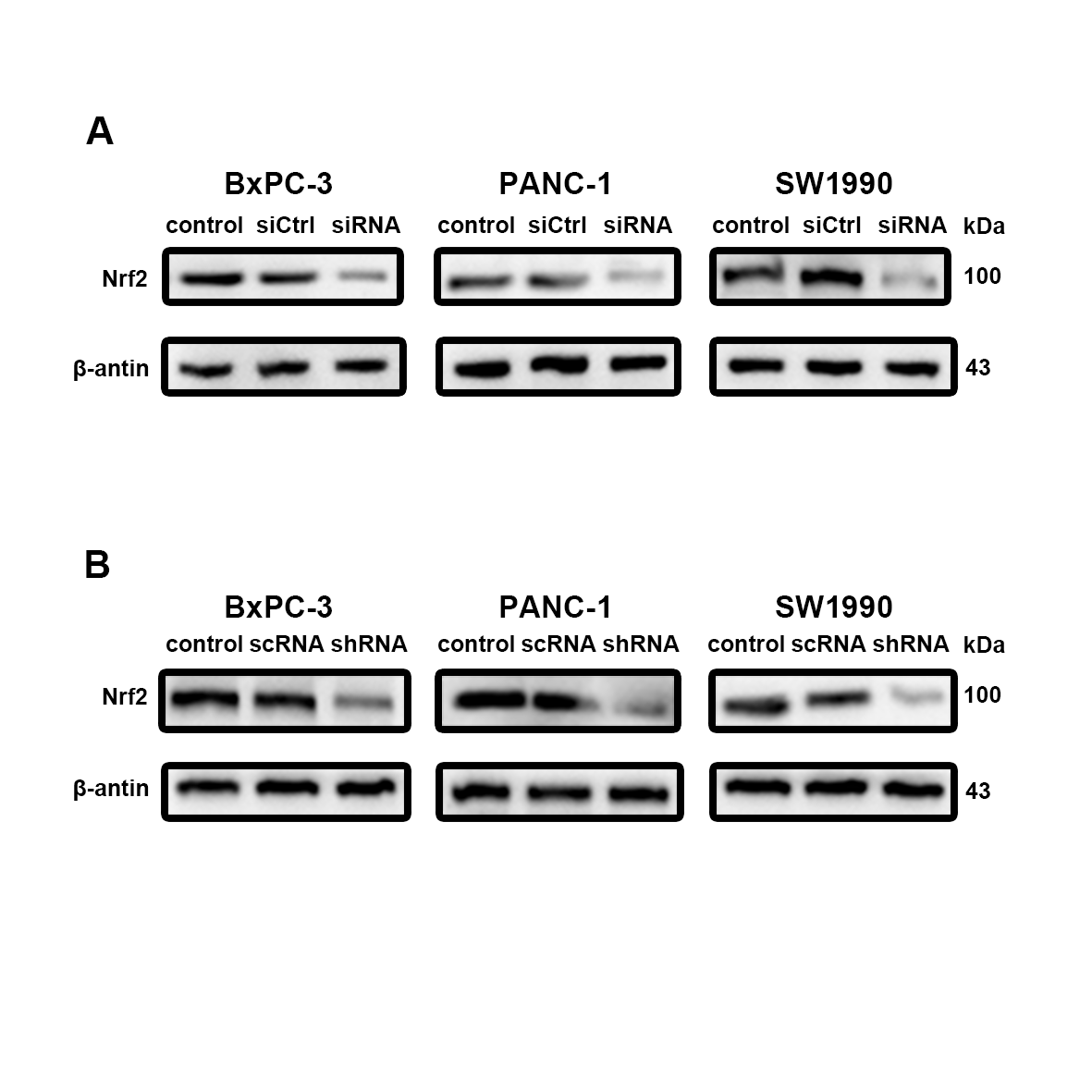

Supplement: Supplementary file 5 — Supplementary figure 4 [file 41419_2020_2938_MOESM5_ESM.tif]
